# Retrograde navigational tunnel technique in peroral endoscopic myotomy for sigmoid-type achalasia

**DOI:** 10.1055/a-2285-2627

**Published:** 2024-04-09

**Authors:** Zhenguo Pan, Zhiying Gao, Zhongshang Sun, Feng Pan

**Affiliations:** 1 Department of Gastroenterology, The Affliated Huaian No.1 People’s Hospital, Nanjing Medical University, Huai’an, China

Standard peroral endoscopic myotomy (POEM) techniques are effective for typical achalasia 1 2 3 4 ; however, limitations are encountered when treating the sigmoid type owing to its complex anatomy. Here, we introduce a novel retrograde navigational tunnel technique in POEM that aims to address these challenges.

A 31-year-old man was admitted to our hospital with a history of postprandial choking sensations for 5 years. Upon admission, a barium meal showed that the esophagus was diffusely dilated with a beak-like appearance at the lower end of the cardia ( Fig. 1 **a** ). We chose to perform POEM after undertaking multidisciplinary consultation and obtaining consent from the patient ( Video 1 ). The procedure was performed with the patient under general anesthesia with endotracheal intubation. A triangular knife was used throughout the surgical procedure. The lower end of the esophagus exhibited a sigmoid contortion and the cardia was seen to be closed ( Fig. 1 **b** ). First, a submucosal injection was administered 30 cm from the incisors to establish the tunnel entrance ( Fig. 2 **a** ). Second, a retrograde submucosal injection was performed from the cardia to the tunnel entrance ( Fig. 2 **b** ). Third, submucosal dissection was performed in the tunnel to navigate from the entrance to 3 cm below the cardia ( Fig. 2 **c,d** ). Both the annular and longitudinal muscles were incised in the tunnel ( Fig. 2 **e** ). Hemostasis was achieved using hot forceps, and the tunnel entrance was closed with metal clamps ( Fig. 2 **f** ). The operation was successfully completed in 47 minutes, without any complications being experienced.

**Fig. 1:**
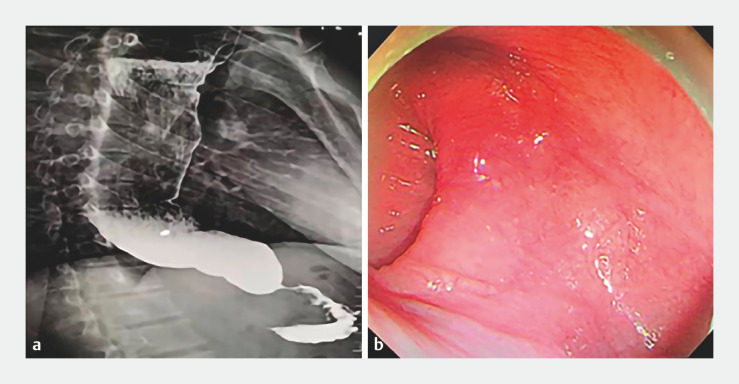
The appearance of sigmoid-type achalasia on: **a** barium swallow, showing a diffusely dilated esophagus with a beak-like appearance at the lower end of the cardia; **b** endoscopic view, showing sigmoid contortion of the lower esophagus and closed cardia.

The navigational tunnel technique is used during peroral endoscopic myotomy for a patient with sigmoid-type achalasia.Video 1

**Fig. 2:**
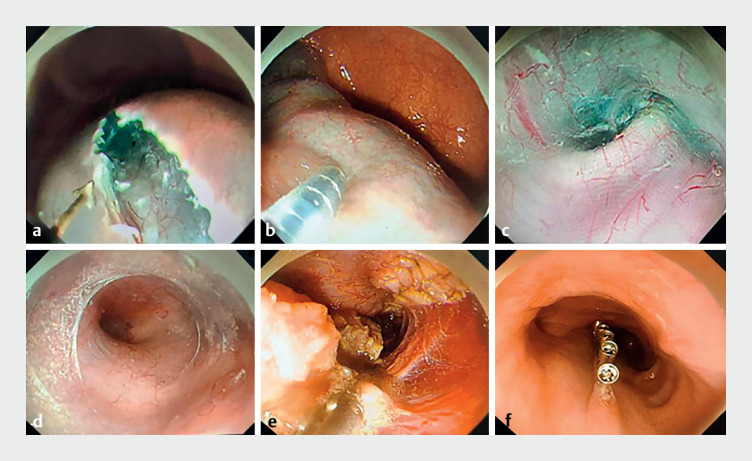
Endoscopic images during the treatment of sigmoid-type achalasia by the navigational tunnel technique for peroral endoscopic myotomy showing: **a** the established tunnel entrance; **b** submucosal injection being performed in retrograde fashion from the cardia to the tunnel entrance; **c** the submucosal dissection navigation route at the flexion; **d** establishment of the submucosal tunnel; **e** incision of the annular and longitudinal muscles; **f** closure of the tunnel entrance with metal clips.

Postoperatively, the patient was fasted and given anti-infection therapy; he was discharged 3 days after the surgery. At 12-month follow-up, the patient had had no recurrence of his choking after eating.

The retrograde navigational tunnel technique in POEM for sigmoid-type achalasia offers two major advantages: (i) reduced surgical time because of continuous submucosal injection; (ii) enhanced accuracy in tunnel navigation, minimizing disorientation during submucosal stripping. In conclusion, the retrograde navigational tunnel technique in POEM is a viable and effective approach for the treatment of sigmoid-type achalasia.

Endoscopy_UCTN_Code_TTT_1AO_2AP

## Notes

Endoscopy E-Videos https://eref.thieme.de/e-videos
*E-Videos* is an open access online section of the journal *Endoscopy* , reporting on interesting cases and new techniques in gastroenterological endoscopy. All papers include a high-quality video and are published with a Creative Commons CC-BY license. Endoscopy E-Videos qualify for HINARI discounts and waivers and eligibility is automatically checked during the submission process. We grant 100% waivers to articles whose corresponding authors are based in Group A countries and 50% waivers to those who are based in Group B countries as classified by Research4Life (see: https://www.research4life.org/access/eligibility/ ). This section has its own submission website at https://mc.manuscriptcentral.com/e-videos .
